# Pathogen- and Type-Specific Changes in Invasive Bacterial Disease Epidemiology during the First Year of the COVID-19 Pandemic in The Netherlands

**DOI:** 10.3390/microorganisms10050972

**Published:** 2022-05-05

**Authors:** Anneke Steens, Mirjam J. Knol, Wieke Freudenburg-de Graaf, Hester E. de Melker, Arie van der Ende, Nina M. van Sorge

**Affiliations:** 1 Centre for Infectious Disease Control, National Institute for Public Health and the Environment (RIVM), 3721 MA Bilthoven, The Netherlands; anneke.steens@rivm.nl (A.S.); mirjam.knol@rivm.nl (M.J.K.); hester.de.melker@rivm.nl (H.E.d.M.); 2 Department of Medical Microbiology and Infection Prevention, Amsterdam UMC, University of Amsterdam, 1105 AZ Amsterdam, The Netherlands; w.freudenburg@amsterdamumc.nl (W.F.-d.G.); a.vanderende@amsterdamumc.nl (A.v.d.E.); 3 Netherlands Reference Laboratory for Bacterial Meningitis, Amsterdam UMC, Location AMC, 1105 AZ Amsterdam, The Netherlands; 4 Amsterdam Institute for Infection and Immunity, 1105 AZ Amsterdam, The Netherlands

**Keywords:** invasive pneumococcal disease, invasive meningococcal disease, *Haemophilus influenzae* disease, serotype, serogroup, molecular epidemiology, COVID-19

## Abstract

COVID-19 control measures have resulted in a decline in invasive bacterial disease caused by *Neisseria meningitidis* (IMD), *Streptococcus pneumoniae* (IPD), and *Haemophilus influenzae* (Hi-D). These species comprise different serogroups and serotypes that impact transmissibility and virulence. We evaluated type- and pathogen-specific changes in invasive bacterial disease epidemiology in the Netherlands during the first year of the SARS-CoV-2 pandemic. Cases were based on nationwide surveillance for five bacterial species with either respiratory (IMD, IPD, Hi-D) or non-respiratory (controls) transmission routes and were compared from the pre-COVID period (April 2015–March 2020) to the first COVID-19 year (April 2020–March 2021). IMD, IPD, and Hi-D cases decreased by 78%, 67%, and 35%, respectively, in the first COVID-19 year compared to the pre-COVID period, although effects differed per age group. Serogroup B-IMD declined by 61%, while serogroup W and Y-IMD decreased >90%. IPD caused by serotypes 7F, 15A, 12F, 33F, and 8 showed the most pronounced decline (≥76%). In contrast to an overall decrease in Hi-D cases, vaccine-preventable serotype b (Hib) increased by 51%. COVID-19 control measures had pathogen- and type-specific effects related to invasive infections. Continued surveillance is critical to monitor potential rebound effects once restriction measures are lifted and transmission is resumed.

## 1. Introduction

Invasive bacterial infections, such as meningitis and sepsis, are associated with high rates of mortality and morbidity worldwide. In the Netherlands, as well as globally, the most common causes of bacterial invasive disease differ per age group and availability and implementation of preventive measures [1]. For meningitis, for example, *Streptococcus agalactiae* and *Escherichia coli* cause nearly 64% of all bacterial meningitis cases in infants (0–89 days of age) in the Netherlands [1], with no vaccines available to prevent these infections. *Neisseria meningitidis*, *Streptococcus pneumoniae*, and *Haemophilus influenzae* are main causes of invasive disease in children under the age of 5 years old and adolescents (for *N. meningitidis*) [1,2,3,4]. *N. meningitidis*, *S. pneumoniae*, and *H. influenzae* are mainly transmitted via the respiratory route. Transmission routes of *S. agalactiae* and *E. coli* differ by age groups; while this may be foodborne, through contact with farm animals [5], or from cash money handling [6], for neonates and infants, transmission mainly occurs vertically (from mother to child) and/or is non-respiratory in the majority of the cases [7,8].

Nationwide surveillance of invasive bacterial infections caused by the aforementioned pathogens allows close monitoring of their incidences and control measures, such as vaccination. In 1993, vaccination against *H. influenzae* type b (Hib) was implemented in the National Immunisation Programme (NIP) [3]. In 2002, vaccination against *N. meningitidis* serogroup C (MenC) was introduced at 14 months, and was accompanied by a catch-up campaign for 1–18-year olds. MenC was replaced by MenACYW in 2018 [9]. In addition, a catch-up campaign among 14–18-year olds with MenACWY was organized and the vaccine has been routinely provided to 14-year olds through the NIP since 2020 [10]. For *S. pneumoniae*, a 7-valent vaccine (PCV7) was implemented in 2006 and replaced by the 10-valent PCV10 in 2011 [11]. The implementation of these vaccines in the routine NIP has dramatically changed the epidemiology of invasive bacterial disease [1,2,3,4], decreasing the incidence of invasive bacterial disease caused by vaccine-covered serotype- and serogroup-specific pathogens in targeted and non-targeted age groups [1,11,12,13].

The occurrence of the COVID-19 pandemic in 2020 resulted in the implementation of public health response measures that were directed at the prevention of respiratory transmission. This included working from home, 1.5 m social distancing, and school and day care closures, which varied in stringency and enforcement, from 15 March 2020 onwards [14]. These control measures did not only limit transmission and subsequent disease caused by SARS-CoV-2, but concomitantly interrupted person-to-person transmission of other pathogens. Indeed, a decline in the incidence of invasive bacterial infections with respiratory transmission including those caused by *N. meningitidis* (IMD), *S. pneumoniae* (IPD) and *H. influenzae* (Hi-D) has been observed in many countries and regions [15,16,17]. In contrast, the incidence of neonatal invasive diseases caused by *S. agalactiae* appears to have been unaffected by the COVID-19-related measures [15]. Indeed, transmission of this pathogen is likely unaffected by the control measures and results from routes other than respiratory. This is also the case for neonatal invasive disease caused by *E. coli*. Therefore, invasive disease cases caused by these (neonatal) pathogens can be used as a control to exclude the impact of the pandemic on isolate submission by microbiology laboratories.

Here, we retrospectively analysed changes in the epidemiology of invasive bacterial diseases in the Netherlands during the first year of the SARS-CoV-2 pandemic, with a specific focus on differences between bacterial species and subtypes. We compared the number of cases and the incidence of invasive disease caused by the respiratory pathogens *N. meningitidis*, *S. pneumoniae*, *H. influenzae*, and the vertically-transmitted pathogens (in infants) *S. agalactiae* and *E. coli* based on nationwide microbiological surveillance from the period April 2015–March 2021.

## 2. Materials and Methods

### 2.1. Data Collection and Patient Information

Nationwide surveillance data were obtained from the Netherlands Reference Laboratory for Bacterial Meningitis (NRLBM) for all studied pathogens. The NRLBM receives bacterial isolates from patients with invasive bacterial disease (isolated from blood and/or cerebrospinal fluid, CSF); isolates are sent by all medical microbiological laboratories in the Netherlands. The invasive bacterial disease surveyed includes IPD for children < 5 years of age, and Hi-D and IMD isolates from patients of all ages. In addition, IPD isolates from patients of all ages are received from nine sentinel laboratories, covering about 25% of the Dutch population. *E. coli* and *S. agalactiae* isolates recovered from blood or cerebrospinal fluid (CSF) are received from patients < 1 years of age. All isolates are typed according to the methods described below. Population statistics (number of inhabitants) were obtained from Statistics Netherlands.

### 2.2. Typing of Bacterial Isolates

The *N. meningitidis* serogroup was determined by Ouchterlony gel diffusion [18]. In case of a culture-negative PCR-positive sample, *N. meningitidis* serogroup was determined by real-time PCR using group-specific probes [19]. *S. pneumoniae* and *H. influenzae* isolates were serotyped by co-agglutination with specific antisera. For *S. pneumoniae*, additional subtyping was assessed by capsular swelling (Quellung reaction) using specific antisera (Statens Serum Institut, Denmark) [20]. *E. coli* isolates were tested for expression of K1 antigen by phage typing according to the manufacturer (SSI Diagnostica) and H- and O-types based on whole-genome sequences [21]. *S. agalactiae* isolates were grouped (Lancefield B) and serotyped through agglutination using specific antisera [22].

### 2.3. Study Period and Patient Categories

For this study, data were extracted from the NRLBM database for the period April 2015 until and including March 2021, and included age, serotype/serogroup and date of sample arrival at the NRLBM. The period April 2020–March 2021 represents the COVID-19 period (the first lockdown in the Netherlands started on 15 March 2020). Data from the preceding five years (April 2015–March 2020) were used as a comparison and were representative of non-COVID-19 years.

For neonatal/infant invasive disease caused by *E. coli* and *S. agalactiae*, data for infants younger than one year were used, since this is resulting from vertical transmission and/or non-respiratory transmission. For *N. meningitidis*, *S. pneumoniae*, and *H. influenzae*, patients were categorized into age groups as appropriate for the epidemiology of the respective pathogens. Specifically, for IPD and Hi-D, age categories included the under-fives, 5–64 years, and older adults (65+). For IMD, the 5–19-year-olds and 20–64-year-olds were analysed separately in addition to the under-five and 65+ age groups. For IPD, the age group 73–79 years was excluded from the analyses to avoid a possible bias due to the introduction of the PPV23 vaccine in autumn 2020 for this cohort.

IPD data were categorized on vaccine type, i.e., PCV10 serotypes (1, 4, 5, 6B, 7F, 9V, 14, 18C, 19F, 23F), PCV13 extra (3, 6A, 19A), PPV23 extra (2, 8, 9N, 10A, 11A, 12F, 15B, 17F, 20, 22F, 33F), and non-vaccine serotypes (all remaining serotypes). Non-typeable isolates and isolates with missing serotype/serogroup information were excluded from the serotype-specific analyses. Specifically, this applied to 0.7% missing or non-typeable serotypes for *S. pneumoniae*, 0.9% missing or ungroupable serogroups for *N. meningitidis*, and 0.1% missing serotypes for *H. influenzae*.

### 2.4. Descriptive Analyses

Analyses were performed separately for the different pathogens and serotypes/serogroups. The percentage change was determined as 1 − (the number of cases in the COVID-19 year/the average number of cases in the five preceding years) × 100. The 5-year moving average per month for the pre-COVID period was based on the targeted month, one month before and one after the targeted month. The incidence was determined by dividing the number of cases in the defined years (April–March) by the number of inhabitants for the same period (for a specific age group)/100,000. The percentage change per serotype/serogroup was determined relative to the number of isolates with known serotype/serogroup. For IPD, results on serotype-specific changes are presented if the serotype had a yearly average of at least 10 cases in the five pre-COVID-19 years. Additionally, ranking was used to determine whether serotypes increased/decreased differentially over time.

## 3. Results

### 3.1. Decrease in Invasive Disease Cases Caused by Respiratory-Transmitted Pathogens in the First COVID-19 Year

**Figure 1:**
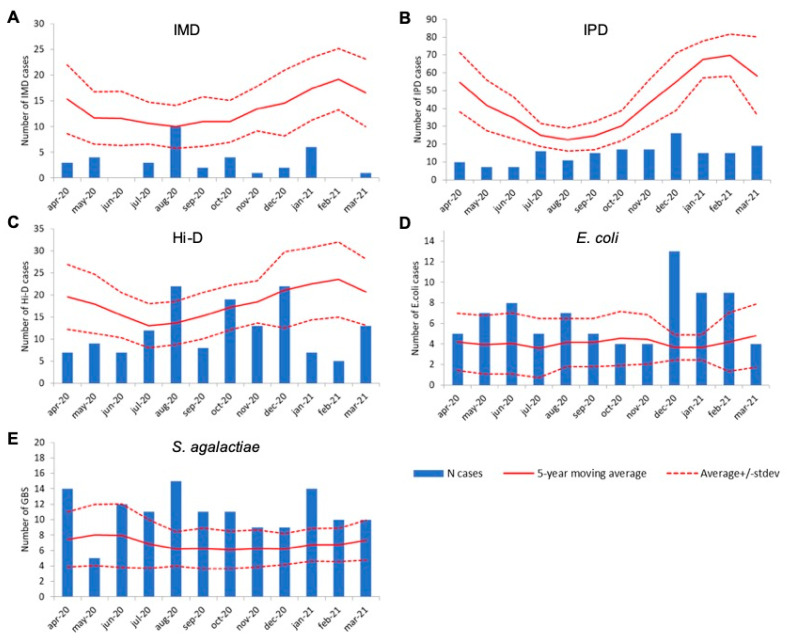
Number of invasive disease cases by month in the first COVID-19 year (2020–2021; bars) based on bacteriological surveillance, the 5-year moving average in pre-COVID years (solid line) and its standard deviation for the pre-COVID period (dashed line) for (**A**): *N. meningitidis* (IMD); (**B**): *S. pneumoniae* (IPD); (**C**): *H. influenzae* (Hi-D); (**D**): *E. coli*; (**E**): *S. agalactiae*. Note that the y-axis differs between pathogens.

Overall, IMD, IPD, and Hi-D decreased by 78% (n = 36 vs. 163), 67% (n = 175 vs. 531) and 35% (n = 144 vs. 221) compared to the average number of cases in the preceding five years 2015–2020 (pre-COVID), respectively. The decrease was immediately apparent in April 2020, which is the first month following lockdown (15 March 2020), for all three pathogens (Figure 1A–C). IMD and IPD case numbers remained lower compared to non-COVID-19 years all throughout the first COVID-19 year (Figure 1A,B). In contrast, Hi-D case numbers were similar or higher compared to the 5-year moving average in August, October, and December 2020 (Figure 1C). Invasive disease cases caused by *S. agalactiae* and *E. coli* showed an increase in the 2015–2020 period, with a similar or increased number of cases in the first COVID-19 year (n = 131 vs. 82 on average and n = 80 vs. 50 on average, respectively; Figure 1D,E).

### 3.2. Age-Specific Effects on Epidemiology of Invasive Disease

We assessed whether the reduction in invasive disease cases was similar across different age groups, despite small numbers. For IMD and IPD, the reduction in invasive disease cases was observed across the different age categories studied; IMD and IPD cases were 58% (n = 8 vs. 17) and 53% (n = 13 vs. 31 pre-COVID) lower for children under the age of five years, and 86% (n = 6 vs. 43) and 69% (n = 89 vs. 283) lower in the 65+ population compared to the pre-COVID average. In contrast, Hi-D cases increased by 13% (n = 38 vs. 34) in the under-fives but decreased by 18% (n = 63 vs. 77) in 5–64-year olds and 61% in the 65+ age group (n = 42 vs. 109). These age-specific differences can be explained by serotype-specific effects, as presented below.

### 3.3. Serogroup-Specific Changes in Invasive Meningococcal Disease Cases

**Figure 2:**
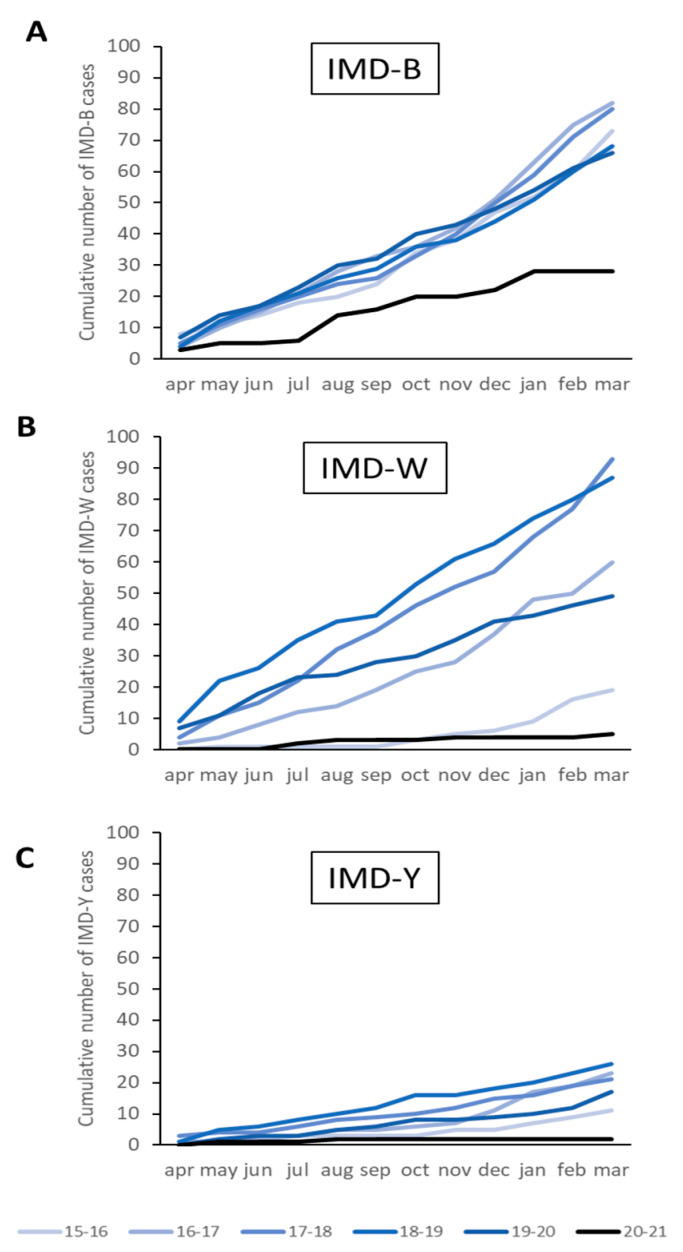
Cumulative number of cases of invasive meningococcal disease caused by (**A**): serogroup B; (**B**): serogroup W; and (**C**): serogroup Y in the first COVID-19 year (2020–2021; black) compared to the previous five pre-COVID-19 years (blue).

Of the 851 *N. meningitidis* isolates received during the study period, 397 (47%) were serogroup B, 29 (3.4%) serogroup C, 313 (37%) serogroup W and 100 (11.8%) serogroup Y. Serogroup A-IMD did not occur in the study period. Serogroup W-IMD and Y-IMD cases declined by 92% (5 vs. 62 on average) and 90% (2 vs. 20 on average), respectively, in the first COVID-19 year compared to the average number of cases in the previous 5 years (Figure 2B,C). These case numbers corresponded to incidences of 0.03/100,000 for serogroup W-IMD and 0.02/100,000 for serogroup Y-IMD in 2020–2021. The decline in serogroup B-IMD cases was less pronounced than for serogroups W and Y, with a 62% reduction in cases (n = 28 vs. 74 on average; Figure 2A), resulting in an incidence of 0.16/100,000 compared to 0.38–0.48/100,000 in the pre-COVID-19 period. Serogroup C-IMD was not detected during the first COVID-19 year.

### 3.4. Serotype-Specific Changes in Invasive Pneumococcal Disease Cases

**Figure 3:**
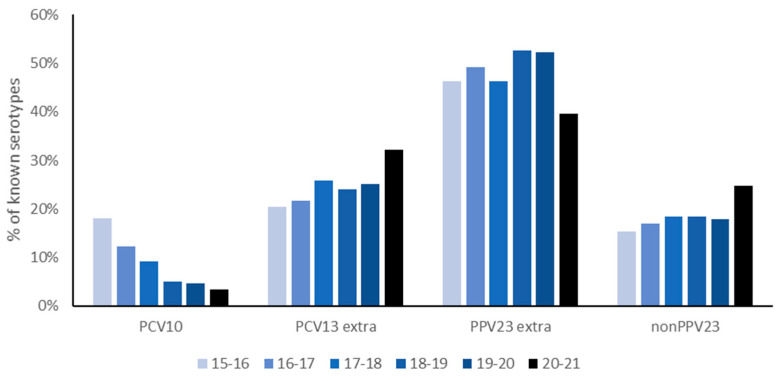
Percentage of IPD cases caused by vaccine serotypes during the years 2015–2021, in all age groups except those aged 73–79 years old.

Overall, 2,830 IPD cases were included in the study. In the five pre-COVID-19 years, an average of 531 IPD isolates per year were submitted by the sentinel laboratories, resulting in an overall incidence of 12.3–14.3/100,000. In the first COVID-19 year, the number of IPD cases decreased to 175 cases (67% reduction), resulting in an overall incidence of 4.3/100,000 among all age groups, excluding the 73–79-year-olds. The proportion of PCV10 serotypes declined over the study period (Figure 3), possibly related to the implementation of PCV10 in the NIP since 2011. Dissecting IPD cases caused by non-PCV10 serotypes in the first COVID-19 year revealed a reduction of 55% in the absolute number of IPD cases (n = 56 vs. 124 on average) caused by the three additional serotypes in PCV13 compared to PCV10 (PCV13 extra) and a reduction of 53% of cases (n = 43 vs. 92 on average) for serotypes not included in PPV23 (non-PPV23) (Figure 3). In contrast, the absolute number of IPD cases caused by additional serotypes in the PPV23 compared to the PCV13 vaccine decreased by 74% (n = 69 vs. 261 on average; Figure 3). Compared to the pre-COVID-19 period, the proportion of cases caused by PCV13extra and non-PPV23 serotypes increased (Figure 3).

We also analysed changes in the number of IPD cases caused by individual pneumococcal serotypes (Table 1). Only serotypes that changed by more than 10% compared to the average change of all-serotype IPD (67%) are discussed. The proportional increase among PCV13extra serotypes was mainly attributable to serotype 19A, which was less affected (50% decrease) than the average IPD decline (67%; Table 1). For PPV23 serotypes, a larger than average decrease was observed for serotypes 8 (76% decrease), 12F (82%), and 33F (87%), as well as for the non-vaccine serotype 15A (86%; Table 1). Of the non-vaccine serotypes, serotype 6C showed the smallest reduction, decreasing by only 16% compared to the average proportion in the previous five years (Table 1). Note, however, that the ranking of the most common serotypes remained stable, with serotypes 19A and 8 being the two most common serotypes for all analysed years, and with serotypes 3, 7F, 22F and 9N remaining in the top 3–6 rank.

### 3.5. Serotype-Specific Changes in H. influenzae Invasive Disease

**Figure 4:**
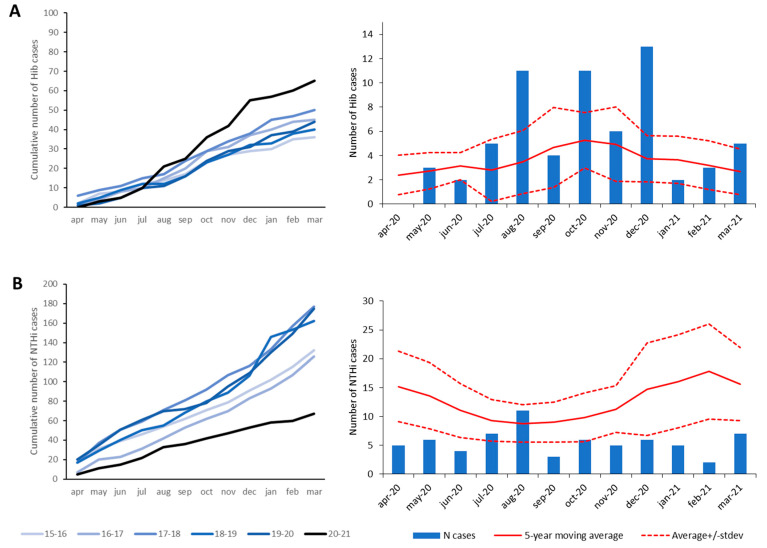
Cumulative number of cases (left) and monthly number of cases (right) for the first COVID-19 year (bars) compared to the 5-year moving average in pre-COVID-19 years (solid line) and the moving average plus/minus its standard deviation for the pre-COVID-19 period (dashed line) for (**A**): *Haemophilus influenzae* serotype b (Hib); and (**B**): non-typeable *H. influenzae* (NTHi).

*H. influenzae* can be categorized in encapsulated (type a, b, c, d, e, f) and non-encapsulated strains, also called non-typeable *H. influenzae* (NTHi). The introduction of glycoconjugate vaccines against Hib in 1993 has resulted in a decline in Hib cases, while the number of invasive disease cases caused by NTHi increased in pre-COVID-19 times [23]. We have focused our analysis on Hib and NTHi, since they cover 90% of all Hi-D cases. Overall, compared to the five preceding years, the number of Hi-D cases decreased by 35% (144 vs. 221 on average) in the first COVID-19 year (Figure 1C). However, there was great disparity between Hib and NTHi cases. The number of Hib cases increased by 51% (65 vs. 43 on average) (Figure 4A) [23], resulting in an incidence of 0.37/100,000 in the first COVID-19 year compared to 0.21–0.29/100,000 in the previous 5-year period. This increase was most pronounced in the age group 5–64 years (97% increase; n = 26), followed by children < 5 years of age (42%; n = 25) and 65+-year-olds (29%; n = 15). In contrast, compared to the pre-COVID-19 period, NTHi cases declined by 57% (n = 67 vs. 154 on average) (Figure 4B), lowering the incidence from 0.73–1.01/100,000 to 0.38/100,000. During the first COVID-19 year, the number of NTHi cases was significantly lower than the 5-year moving average during all months except in August (Figure 4B), whereas Hib cases were higher than the 5-year moving average for nearly all months (Figure 4A).

## 4. Discussion

In the present study, we evaluated the effect of the implementation of public health measures in response to the COVID-19 pandemic by analysing epidemiological changes in the occurrence of invasive disease caused by different bacterial pathogens. We observed a 35% to 78% reduction in the incidence of invasive disease caused by *N. meningitidis*, *S. pneumoniae* and *H. influenzae*. In contrast, no decrease but a slight increase in infant invasive disease cases caused by *S. agalactiae* and *E. coli* was observed in the COVID-19 year compared to the 2015–2020 pre-COVID-19 period. This trend is consistent with the observed trend over a longer period and may be related to better symptom recognition, increased testing and reporting, and a genetic shift within the *S. agalactiae* population resulting in increased virulence [24,25]. Overall, the steady or increasing number of cases for these neonatal pathogens suggests that the national surveillance of invasive bacterial diseases, which relies on voluntary isolate submission from clinical diagnostics laboratories, was generally not disrupted. However, we cannot fully exclude the possibility that the surveillance was affected by the care and mass diagnostics of COVID-19 patients, or that specific age groups were differentially affected, as we were not able to estimate possible age-specific changes in our control group [26]. Because of the surveillance system itself, with subtyping being conducted by the reference laboratory after isolate submission, it is unlikely that potential effects on surveillance would differ between sub-types.

A decrease in the occurrence of IMD during the COVID-19 pandemic has been observed by many countries [15,17]. For the results presented here, it is important to note that the MenACWY vaccine has been implemented in the Netherlands in 2018 [9]. Consequently, the decrease in IMD cases caused by serotypes W and Y are partially influenced by the protective effect from MenACWY vaccination [10]. The decrease in MenB cases by 62% therefore most accurately reflects the impact of COVID-19 on IMD, since IMD-B disease is not affected by a recent introduction of a vaccine.

Changes in IPD were different for different serotypes. Specifically, serotypes 8, 12F, 33F (all in PPV23 but not in PCV10) and 15A (non-vaccine type) decreased more than other serotypes. In Taiwan, a similarly large decrease in serotype 15A was observed [27]. Serotypes 8, 12F, and 15A have been increasing in Europe since before the COVID-19 pandemic and are known to have a high invasive capacity [28,29,30,31,32]. Generally, these serotypes are dominant in younger individuals and those without underlying medical conditions [28]. Unfortunately, we lack information on underlying medical risk conditions in the study cohort. In contrast to the abovementioned serotypes, serotypes 19A and 6C showed a smaller decrease among IPD cases. This is of interest since these serotypes have become more common in pre-COVID years in several countries that use PCV10 [33], likely as a result of serotype replacement [34]. Whether the relatively limited decrease can be partly explained by an offset caused by serotype replacement after PCV10 introduction is yet unknown [35].

Our serotype-specific observations differ from reports by some other countries. In Germany, the proportion of vaccine serotypes (PCV13, PCV15, PCV20) remained constant during the COVID-19 control measures, although 12F and 22F seemed to represent a smaller proportion in 2021 than in earlier years, indicating a larger decrease during COVID-19 times and “other serotypes” comprised a larger proportion in 2021 [36]. In Taiwan, they noticed a large decrease in 19A in contrast to our modest reduction in this serotype [27]. Finally, in Switzerland, no serotype-specific patterns were observed during the period with COVID-19 control measures, although serotype 23B increased more than other serotypes after easing the non-pharmaceutical interventions [35]. Serotype-specific changes may reflect age-specific (adherence to) non-pharmaceutical interventions. Furthermore, the serotype-specific changes may reflect differences in the propensity of these serotypes to cause secondary infections following respiratory viral infections. Indeed, there were large decreases in overall viral infections during the COVID-19 period, while carriage patterns were reported not to be affected during the COVID-19 pandemic [37].

For Hi-D, there was a discrepancy in the changes during the COVID-19 year, which were attributed to the different behaviour of serotypes. NTHi disease cases decreased across all age groups, whereas Hib increased in under-fives and 5–64-year-olds (specifically in 50–64-year-olds) but decreased in the 65+ population. The Hib increase during the first COVID-19 year is striking as preventive measures against COVID-19 were in place and diseases caused by other respiratory-transmitted pathogens decreased. Although not the focus of this study, the vaccine effectiveness against Hib disease did not seem to be different from previous years [23]. We are currently investigating the possible cause of this increase in Hib disease.

Overall, we showed that the changes in epidemiology during the first COVID-19 year differed between pathogens, subtypes and age groups, with the largest discrepancy seen for Hib and NTHi. Our study indicates that, overall, the disease burden of respiratory bacterial pathogens was decreased by the preventive measures against COVID-19, but it also shows that other factors affected the epidemiology simultaneously; for IMD, the recent MenACWY vaccination campaign likely played a role in the observed differences between serogroup B versus serogroup W and Y IMD. For Hib, unknown factors seem to have affected the epidemiology [23]. The differences in pneumococcal serotype-specific changes might be associated with the epidemic potential, invasive capacity, and/or propensity of the different serotypes to cause secondary infections. Possible differences by risk group and age in (adherence to) control measures may have played a role in causing different decreases for different age groups. Altogether, we stress the importance of subtype-specific surveillance during the COVID-19 pandemic and specifically when measures are relaxed again, given the worry that the interruption of transmission has created a larger group of susceptible individuals [38].

## Figures and Tables

**Table 1:** Number of cases of invasive pneumococcal disease per serotype as average and range of pre-COVID-19 period (2015–2020) and first COVID-19 year in all age groups except those aged 73–79 years old. Decrease in number of serotype-specific IPD cases is indicated by percentage. * If covered by PCV10, then PCV10 is used, if not in PCV10 but in PCV13, then PCV13 is used, if not in PCV13 and PCV10 but in PPV23, then PCV23 is used. If not covered by any of the currently available vaccines, non-vaccine type (NVT) is used.

Serotype	Covered by Vaccine *	Average Annual Number (Range) of Cases, April 2015–March 2020	Number of Cases in First COVID-19 Year (Range), April 2020–March 2021	Decrease in COVID-19 versus Non-COVID-19 Year (%)
All serotypes		531 (503–576)	175	67%
8	PPV23	126 (118–129)	30	76%
19A	PCV13	76 (73–83)	38	50%
3	PCV13	46 (38–59)	18	61%
22F	PPV23	35 (27–39)	11	68%
9N	PPV23	28 (21–34)	9	68%
12F	PPV23	23 (19–27)	4	82%
7F	PCV10	21 (4–43)	0	100%
6C	NVT	18 (16–22)	15	16%
33F	PPV23	16 (11–20)	2	87%
15A	NVT	14 (12–15)	2	86%
23B	NVT	13 (10–16)	5	60%
23A	NVT	11 (6–15)	4	64%
1	PCV10	11 (0–30)	0	100%
10A	PPV23	11 (8–14)	4	62%

## Data Availability

The data presented in the study are available on request with the corresponding author but are not publicly available due to regulations in the Personal Data Protection Act.
